# Contribution of Epithelial and Gut Microbiome Inflammatory Biomarkers to the Improvement of Colorectal Cancer Patients’ Stratification

**DOI:** 10.3389/fonc.2021.811486

**Published:** 2022-02-07

**Authors:** Elena Ionica, Gisela Gaina, Mihaela Tica, Mariana-Carmen Chifiriuc, Gratiela Gradisteanu-Pircalabioru

**Affiliations:** ^1^ Department of Biochemistry and Molecular Biology, Faculty of Biology, University of Bucharest, Bucharest, Romania; ^2^ Laboratory of Cell Biology, Neuroscience and Experimental Miology, Victor Babes National Institute of Pathology, Bucharest, Romania; ^3^ Bucharest Emergency University Hospital, Bucharest, Romania; ^4^ Biological Science Division, Romanian Academy of Sciences, Bucharest, Romania; ^5^ Research Institute of the University of Bucharest, University of Bucharest, Bucharest, Romania

**Keywords:** colorectal cancer, inflammation, gut microbiota, biomarkers, patients’ stratification

## Abstract

In order to ensure that primary endpoints of clinical studies are attained, the patients’ stratification is an important aspect. Selection criteria include age, gender, and also specific biomarkers, such as inflammation scores. These criteria are not sufficient to achieve a straightforward selection, however, in case of multifactorial diseases, with unknown or partially identified mechanisms, occasionally including host factors, and the microbiome. In these cases, the efficacy of interventions is difficult to predict, and as a result, the selection of subjects is often random. Colorectal cancer (CRC) is a highly heterogeneous disease, with variable clinical features, outcomes, and response to therapy; the CRC onset and progress involves multiple sequential steps with accumulation of genetic alterations, namely, mutations, gene amplification, and epigenetic changes. The gut microbes, either eubiotic or dysbiotic, could influence the CRC evolution through a complex and versatile crosstalk with the intestinal and immune cells, permanently changing the tumor microenvironment. There have been significant advances in the development of personalized approaches for CRC screening, treatment, and potential prevention. Advances in molecular techniques bring new criteria for patients’ stratification—mutational analysis at the time of diagnosis to guide treatment, for example. Gut microbiome has emerged as the main trigger of gut mucosal homeostasis. This may impact cancer susceptibility through maintenance of the epithelial/mucus barrier and production of protective metabolites, such as short-chain fatty acids (SCFAs) *via* interactions with the hosts’ diet and metabolism. Microbiome dysbiosis leads to the enrichment of cancer-promoting bacterial populations, loss of protective populations or maintaining an inflammatory chronic state, all of which contribute to the development and progression of CRC. Meanwhile, variations in patient responses to anti-cancer immuno- and chemotherapies were also linked to inter-individual differences in intestine microbiomes. The authors aim to highlight the contribution of epithelial and gut microbiome inflammatory biomarkers in the improvement of CRC patients’ stratification towards a personalized approach of early diagnosis and treatment.

## 1 Introduction

Noncommunicable diseases (NCDs) are now responsible for most of the global deaths, among which cancer is a serious health problem in all countries, regardless of socio-economic status. According to data provided by the World Health Organization (WHO) in 2020, at a global level one in five people faces a cancer diagnosis during their lifetime. The most important actions to increase life expectancy should be increasing prevention, diagnosis, treatment and management, palliative care, and surveillance. According to the Agency for Research on Cancer, the disease is the first or second leading cause of death in 2019 in the USA, while ranking third or fourth in an additional 22 countries. An estimated 18.1 million people around the world (excluding nonmelanoma skin cancer) developed cancer, out of which 9.9 million died from the disease before age 70 in 112 of 183 countries (1, 2). Lung cancer (11.6% of all cases), followed by female breast (11.6%) and colorectal (CRC) cancers (10.2%) are the most frequently diagnosed cancers (3).

Although developed countries have the highest CRC incidence and mortality rates worldwide, CRC incidence has recently started increasing in low-income and middle-income nations (2, 4). This reflects changes in lifestyle factors and diet, an increased intake of animal-source foods, a more sedentary lifestyle, leading to decreased physical activity and increased prevalence of excess body weight (1). However, complex causes reflect both aging and global population growth, and also changes in the prevalence and distribution of the main risk factors for cancer. In some cases, these factors were associated with socioeconomic development (5). It has been observed that in countries undergoing major transitions, such as countries from Eastern Europe, South Europe, South Central Asia, and South America, the incidence rate tends to increase uniformly with the Human Development Index (HDI) (1). Therefore, primary prevention remains the most important strategy to reduce the global prevalence of colorectal cancer.

In 2020, CRC ranks third in terms of incidence and second in terms of mortality (1, 5). Even in countries where national CRC screening programs exist the mortality is around 30%, and only a small proportion of CRCs are diagnosed through population-based screening programs (1, 5).

Before the year 2000, the changes in pattern behavioral risk factors (smoking reduction, change in dietary pattern) and also increasing number of CRC screening programs around the world (SUA, UK, Switzerland, Austria) was associated with a decreased rate of CRC incidence. However, there is still a gap in diagnosing individuals at a localized stage, probably because screening programs include only patients older than 50 years and only track behavioral changes. The screening is based on colonoscopy, a method that predominantly prevents tumor metastasis by removing premalignant polyps (6).

After 2000, due to rapid technological improvement and affordable costs of molecular techniques, scientists were able to define criteria for CRC patients’ stratification based on sex, age, family history, genetic susceptibility, and endoscopy and colonoscopy examinations. Consequently, the individualized treatment has been achieved more rapidly. In 2015, in a joint effort to understand the heterogeneous clinical and drug outcomes observed in CRC patients, a consortium of researchers defined a new criterion for CRC patients’ stratification based on Consensus Molecular Subtyping (CMS). This highlighted the interconnection between six classification systems that were finally grouped into four molecular subtypes. The first subtype (CMS1) is based on microsatellite instability (MSI), significance of immune activation and hypermethylation, specific processes for the early-stage sporadic colorectal cancer. For early diagnosis and treatment of CRC patients, the MSI model (CMS1) has become a key biomarker due to advances in the understanding of the immune microenvironment of colorectal cancer. CRC patients with different microsatellite statuses exhibited different compositions and distributions of immune cells and cytokines within their tumor microenvironments (TMEs). A complex crosstalk between different regulatory pathways was observed in the TME, with a key role in the occurrence, progression, and treatment of tumours, representing an important source of potential therapeutic targets. Intestinal microbiome (IM) has emerged as the main environmental trigger of intestinal mucosal homeostasis that may also influence cancer susceptibility through maintenance of the epithelial/mucus barrier and production of protective metabolites *via* interactions with host diet and metabolism. Dysbiosis leads to the enrichment of cancer-promoting bacterial populations, loss of protective populations, development, and progression of CRC by maintaining an inflammatory chronic state, etc., while variations in patient responses to cancer immuno- and chemotherapies have been linked to inter-individual differences in intestine microbiomes (7–9). Accumulating evidence suggests that chronic inflammation and the metabolites accumulated ensuing inflammation contribute to tumor initiation and tumor progression (10–12).

In this article, we aim to focus on the complex network signaling pathway crosstalk between colonocytes, microbiome, and tumor cells at the earlier-stage sporadic colorectal cancer, and to reveal the role of inflammatory biomarkers in CRC screening programs.

## 2 Advances in the Molecular Stratification of Colorectal Cancer

CRC is a highly heterogeneous disease, with varying clinical outcomes, morphological features, and genetic and gene regulatory levels which contribute to differences in individual response to therapy. The histopathological parameters typically used for diagnosis are not enough to recognize high risk patients, while molecular features are still sparsely used in current clinical practice.

Explosion in knowledge regarding genetic, epigenetic, and biochemical alterations, associated with the evolution of CRC, correlated with advances in molecular biology, sequencing, computer science and modern bioinformatics have facilitated the development of different patients’ stratification strategies. A systematic review of literature on developed stratification models on CRC indicates that current screening guidelines for CRC stratify patients based on different criteria as presented in **Table 1**.

**Table 1:** CRC patients’ stratification criteria.

Criteria	References
Human Development Index	(13)
Common genetic susceptibility loci	(14, 15)
Lifestyle and environmental factors	(16)
Integration of personalized patient-derived organoids drug screening and patient-derived xenografts generation	(17–19)
DNA sequencing of archival or fresh tumor biopsy	(19–21)
Consensus molecular subtypes (CMS)	(22–25)
Personalized patient—derived tumor organoids drug screening and patient-derived xenografts	(17)
Current screening guidelines based on age and family history	(1, 26)
Screening based on lifestyle, environmental and genetic factors	(16)
Immunoscore	(27)
Molecular matching with predetermined monotherapy (PREDICT trial)	(28)
Fresh biopsy-derived DNA sequencing (WINTHER trial)	(29)
Genomic and transcriptomic analysis (WIN trial)	(29)
Individual molecular alteration (NCI-MATCH trial)	(30)
Genomic instability	(31)

As shown in **Table 1**, selection criteria for the enrolment of prospective subjects include age, gender, molecular signature, inflammation scores, or Dietary Inflammatory Index (DII^®^). The DII, a literature-derived population-based dietary index, was analyzed by Shivappa in nine studies that examined the association between the diets’ inflammatory potential and CRC. In their meta-analysis they demonstrate that limiting the consumption of pro-inflammatory foods, such as red meat, and increasing consumption of anti-inflammatory nutrients may play an important role in reducing the risk of CRC. Anti-inflammatory foods include fiber, monounsaturated fatty acids, polyunsaturated fatty acids, omega 3, omega 6, vitamins and minerals, all associated with a lower DII score. While pro-inflammatory foods include carbohydrates, proteins, trans fat, total fat, cholesterol, saturated fatty acids, iron, etc., associated with a higher DII score (32, 33). Evidence suggests that modifiable lifestyle factors, namely, excess adiposity, poor diet, and physical inactivity play an important role in the occurrence and progression of this disease (34). While for diseases with known underlying mechanism or targeted therapy, the selection of subjects can be straightforward to obtain high efficacy of a given intervention, this cannot be achieved, unfortunately, in the case of CRC. It is a multifactorial disease, with unknown or partially elucidated mechanisms, sometimes including host factors, local environmental factors, lifestyle factors (35), microbiome, and host diets that have been extensively studied. All of these play a causal and protective role in the development of CRC (36). Based on 19 factors, Jeon and col. created a risk score that summarizes an individuals’ overall lifestyle and environment CRC risk profile by using data from 14 population-based studies. They demonstrate that both lifestyle, environmental factors, and common genome-wide association study (GWAS) variants are independent risk predictors for CRC (16).

Advances in molecular techniques bring new criteria for patients’ stratification, e.g., mutational analysis at the time of diagnosis to guide treatment. By reviewing the literature, Coleman and co. found that the caloric content and composition of diets are linked to the development of obesity and were associated with persistent and transmissible alterations in the IM. It was suggested that differences in diet and in the IM may be responsible for variations in CRC prevalence between two similar human populations (37).

The CRC onset and progress involves multiple sequential steps with accumulation of genetic alterations including mutations, gene amplification, and epigenetic changes. Although inherited genetic susceptibility has a key role in a subset of CRC cases, in general, CRC cases are sporadic and non-inherited. Traditionally, CRC can be divided into familial CRC (hereditary CRC, HCRC) and sporadic CRC (non-hereditary CRC, NHCRC) (38).

Other than HCRC which accounts for approximately 20–25% of all types of CRC, as determined by non-modifiable risk factors such as familial history of CRC, adenomatous polyps, specific mutations occurring in genes involved in the tumorigenic pathway, an approximate of 75% of CRC tumors have some genetic defects that occur throughout life, stemming from a multitude of factors.

Even if new molecular pathways, critical for tumor development, are continuously discovered and their molecular targeting is a success in the experimental models, the number of effective therapies for CRC patients is still very limited.

While in the early 1990s, researchers were able to link the mutations of some genes to the susceptibility of CRC and postulated the idea that CRC progression is correlated with the accumulation of genetic changes, more recently there has been an increased emphasis on the heterogeneity of colon cancer, and the involvement of the intratumoral environment (39–43).

The two main pathways used to classify CRC and guide the personalized therapeutic strategy are the chromosomal instability pathway (CIN) that accounts for 85% of cases, and the microsatellite instability pathway (MSI) that represents 15% of total CRC cases (44).

Initially, this classification was used for dichotomization into good and poor prognosis patients, whereas current research reveals that separation into strictly two groups does not reflect the biological diversity specific to colon cancer (45).

Most of the sporadic CRC cases are explained using *CIN model*, which suggests a predictable progression with sequential accumulation of mutations in specific genes like adenomatous polyposis coli (APC), WNT, etc. The model provides signs that can be used for risk assessment, early detection, prognosis, and treatment of the disease. According to this model, the sequential accumulation of mutations that eventually leads to CRC provides a level of opportunity to prevent CRC before these mutations reach a threshold level.

The *MSI model* represents a class of colon cancer characterised by high mutational load caused by defective DNA mismatch repair (MMR). The microsatellites, representing repetitive DNA sequences, are especially sensitive to mutation due to dysfunctional MMR and a high abundance of microsatellite length alteration. The detection of MSI is identified *via* a PCR-based assay categorizing tumours as either MSI-high (MSI-H), MSI-low (MSI-L) or microsatellite stable (MSS), based on the number of microsatellite markers demonstrating instability. In sporadic MSI tumors, defective DNA MMR usually result from CpG (5’-CG-3’) island promoter methylation and therefore the inactivation of the MLH1 gene which encodes a key gene in MMR pathway. This promoter methylation is accompanied by other methylation processes of promoters throughout the genome. This phenomenon is known as CpG island methylator phenotype (CIMP). Testing for CIMP is performed *via* PCR for hypermethylation in CACNA1G, MLH1, NEUROG1, RUNX3, and SOCS1 (46). Based on MSI status, an international consortium consolidated separate findings into one overarching stratification system, the Consensus Molecular Subtypes (CMS) of colorectal cancer (22).

As mentioned, a consortium of researchers highlighted the interconnection between six classification systems that were finally grouped into four molecular subtypes (CMS): CMS1 (MSI Immune) (14% of classified molecular clusters), microsatellite instability, significance of immune activation and hypermethylation; CMS2 (Canonical) (37%), shows changes in the WNT and MYC signalling pathways in epithelial cells; CMS3 (Metabolic) (13%), presents disorders in the signalling pathways in epithelial cells and metabolic changes; and the CMS4 subtype (Mesenchymal) (23%) shows changes in mesenchymal–epithelial cells, prominent stromal invasion and angiogenesis, changes that are accompanied by activation of growth factor β (TGF-β) (22, 47). The remaining 13% of the analyzed molecular clusters could not be included in any subtype because the characteristics are specific to several subtypes; it is considered to have a phenotype of transition from one cluster to another. This classification is the most comprehensive, preserving the relation between specific molecular changes, biological classification, and clinical classification.

## 3 Tumor Microenvironment Heterogeneity as a Potential Source of CRC Biomarkers and Therapeutic Targets

Over the last 10–15 years, the CRC research field has moved from mainly assessing mutations to measuring gene expression patterns, in order to describe a broader spectrum of CRC heterogeneity (48). The molecular heterogeneity involves several molecular pathways and molecular changes unique to a tumor of an individual and, although the existence of heterogeneity in CRC has been recognized for a longer period, it is sparingly incorporated as a determining factor in current clinical practice.

Development of TME is driven by genetic instability of cancer cells and by epigenetic factors in response to endogenous stress stimuli or exogenous factors like pH changes, aberrant angiogenesis, hypoxia, glucose deprivation, acidosis, and oxidative stress. Endogenous stressors are associated with imbalanced cell growth, increased mutation rate, errors in lipid and glycoprotein biosynthesis and decreased amino acid supplies (49, 50).

The tumor stromal-inflammatory interface represents a dynamic space which includes growth factors (hepatocyte growth factor (HGF), insulin-like growth factors (IGF), nerve growth factor (NGF), wingless-type MMTV integration site family member 1 (WNT1), epidermal growth factor (EGF), fibroblast growth factor 2 (FGF2), vascular endothelial growth factor (VEGF), platelet derived growth factor (PDGF).), cytokines (IL-1, IL-6, IL-8), chemokines (CXC chemokines CXCL1 to CXCL12, CC chemokines CCL2 and CCL20), enzymes (LOX family oxidases and LOX-like proteins 1–4, COX2), matrix metalloproteinases (MMP-1, MMP-2, MMP-7, MMP-9, MMP-13, and MMP-14), ECM proteins (fibronectin, collagen I and III, EDA-fibronectin, tenascin C, and SPARC), and metabolic intermediates that are secreted during the exchanges (between various molecular information) associated with stromal and cancer cells transitions. Recruitment, activation, reprogramming and persistence of inflammatory and stromal cells in the extracellular space are the consequences of a reciprocal relationship between TME and cancer cells (51). Studies have shown that infiltration of immune cells into TME is an important factor affecting tumor heterogeneity and prognosis, and infiltrated immune cells and cytokines secreted by the inflammatory process may play dual roles by inhibiting or promoting tumors.

In light of all these, the above mentioned MSI model (CMS1) is of paramount importance for the early diagnosis and treatment of CRC patients due to advances in the understanding of the immune microenvironment of colorectal cancer. CRC patients with different microsatellite statuses present different compositions and distributions of immune cells and cytokines within their TMEs (52).

A complex crosstalk between different regulatory pathways was observed in the TME, which played a key role in the occurrence, progression, and treatment of tumours, representing an important potential therapeutic target (53). As so, the TME is permanently remodeling and reprogramming according to the modification of numerous physical, biochemical, and stromal cells functions like pH, oxidative stress, ECM stiffness, metabolism, inflammation, and immunity. Therefore, inhibition of one specific target leads to different answers, at different levels in different regulatory pathways. In addition, the cell is able to avoid the path that is blocked so that the entire signaling network functions; cancer cells develop new adaptative strategies to adjust their phenotype to the variation of TME conditions (50). New developed heterogenous cancer cells subpopulations appear and show different characteristics in terms of plasticity, stem-like phenotype, metabolic reprograming, invasion, immunosuppression, and therapeutic resistance (51). According to Alsibai, the most widely known plasticity processes are the epithelial–mesenchymal transition (EMT) and the reversible mesenchymal–epithelial transition (MET). Cancer cells follow similar EMT processes to establish metastases. EMT facilitates epithelial cancer cells to enter a mesenchymal-like state by endowing the migratory and invasive properties, which enables a primary tumor to move and colonize distant organs and form metastases. This is a critical step in early phase of cancer metastasis and is closely linked to carcinogenesis, invasion, recurrence, and therapy resistance (50). One of the goals of the researchers for stratifying patients according to the specific crosstalk patterns is to analyze how they communicate with the main signaling pathways involved in carcinogenesis, as well as to show the nature of the relationship between different subtypes of tumor cells and protein expression.

## 4 Contribution of Gut Microbiota Derived Markers to CRC Patients’ Stratification

Nearly all the digestion and absorption of nutrients is carried out in the gastrointestinal tract (GI). Abundant gut microbes in the GI, estimated to total about 10^13^–10^14^ CFU (colony forming units)/ml, are involved in the process. Bacterial products together with food residue make up most of the contents in the intestine and are directly or indirectly in contact with the mucus layer separating them from the epithelial cells. The gut microbiome, the largest micro-ecosystem in the human body, is symbiotic with the host and maintains normal physiological processes in a dynamic equilibrium state (54). It establishes a complex interaction with host cells and even a small disturbance may lead to the breakdown of intestinal homeostasis just like the ‘butterfly effect’ (55). The maintenance of this precise balance requires the control of epithelial cells *via* different immune mechanisms and significantly contributes to the constitution of the intestinal immune barrier (56). It is widely acknowledged that the gut microbiome has an important role in the development of a properly functioning mucosal and systemic immune system. The intestinal microbes, either eubiotic or dysbiotic, could influence the CRC evolution, through a complex and versatile crosstalk with the intestinal and immune cells, permanently changing the tumor microenvironment.

With the rapid development of molecular biology, genomics, bioinformatic analysis technology, researchers linked CRC to intestinal dysbiosis (57, 58), and to specific microbial composition, structure, and function signatures (59).

Intestinal dysbiosis leads to the enrichment of cancer-promoting bacterial populations, loss of protective populations, development, and progression of CRC by maintaining an inflammatory chronic state etc., while variations in patient responses to cancer immuno- and chemotherapies were linked to inter-individual differences in gut microbiome.

However, despite long-standing associations between diet, the microbiome, and CRC (60, 61), the specific mechanisms by which the gut microbiome may influence not only the starting events of carcinogenesis, but also its progression, have only been highlighted recently (8, 9, 54). Several studies investigating the CRC microbiota have shown that the dysbiosis associated with CRC is characterized by a relatively decreased abundance of obligate anaerobes, an increase in potential pathogenic bacteria (pathobionts), and a reduction of butyrate-producing bacteria (62), such as *Bifidobacteria*, *Lactobacillus*, and *Bacteroides*. Molecular fingerprinting and clone sequencing methods revealed that the microbiota of subjects with adenomas harboured a higher proportion of *Proteobacteria* and lower abundance of *Bacteroidetes* when compared to that of control subjects (63). These initial findings were later confirmed in a study that used 16S rRNA gene amplicon 454 pyrosequencing methods to characterize the microbiome. Sanapareddy et al. (13) found an enrichment of potential pathogens like *Helicobacter*, *Bacteroides fragilis*, *Fusobacterium nucleatum*, *Acinetobacter*, *Pseudomonas*, *Escherichia coli*, and other *Proteobacteria* in mucosal biopsies of adenoma patients compared to non-adenoma controls (64).

Brim et al. (14) analyzed the faecal microbiota of African American patients with colorectal adenomas and reported altered microbial changes between adenoma patients and healthy controls (65). Using experimental models of CRC, Wei et al. (15) showed that precancerous lesions had dysbiosis associated with an increased abundance of *Allobaculum* spp. and *Ruminococcus obeum* (66). These data suggest that changes in the adherent microbial community composition may have a role in the development of adenomas. Recently, Yachida et al. (67) conducted the largest metagenomics (*n* = 616) and metabolomics (*n* = 406) analysis on human CRC. Specific shifts in microbiome composition, metabolome and bacterial gene abundance were correlated with different stages of CRC progression. Members of the *Firmicutes*, *Fusobacteria*, and *Bacteroidetes* phyla were increased in carcinoma patients compared to healthy controls and adenomas. Moreover, early stages of disease were characterised by an enrichment of taxa such as *Actinomyces odontolyticus*, *Atopobium parvulum*, *Phascolarctobacterium succinatutens*, and *Desulfovibrio longreachensis*. Lactic acid-producing bacteria such as *Bifidobacterium animalis*, *Streptococcus thermophiles*, and *Streptococcus mutans* were prevalent only in the healthy control group. Progression from healthy to advanced adenoma was characterized by a significant increase of *Bacteroides* species such as *B. dorei* and *B. massiliensis* whereas an increase in *B. massiliensis*, *B. ovatus*, *B. vulgatus*, and *E. coli* was reported for the progression from advanced adenoma to carcinoma (68).

The pathogenic bacteria prevalent in the CRC patients’ microbiome can secrete toxic metabolites that affect intestinal epithelial cells and cause a chronic inflammatory response, which contributes to the development of CRC (69, 70).

**Figure 1:**
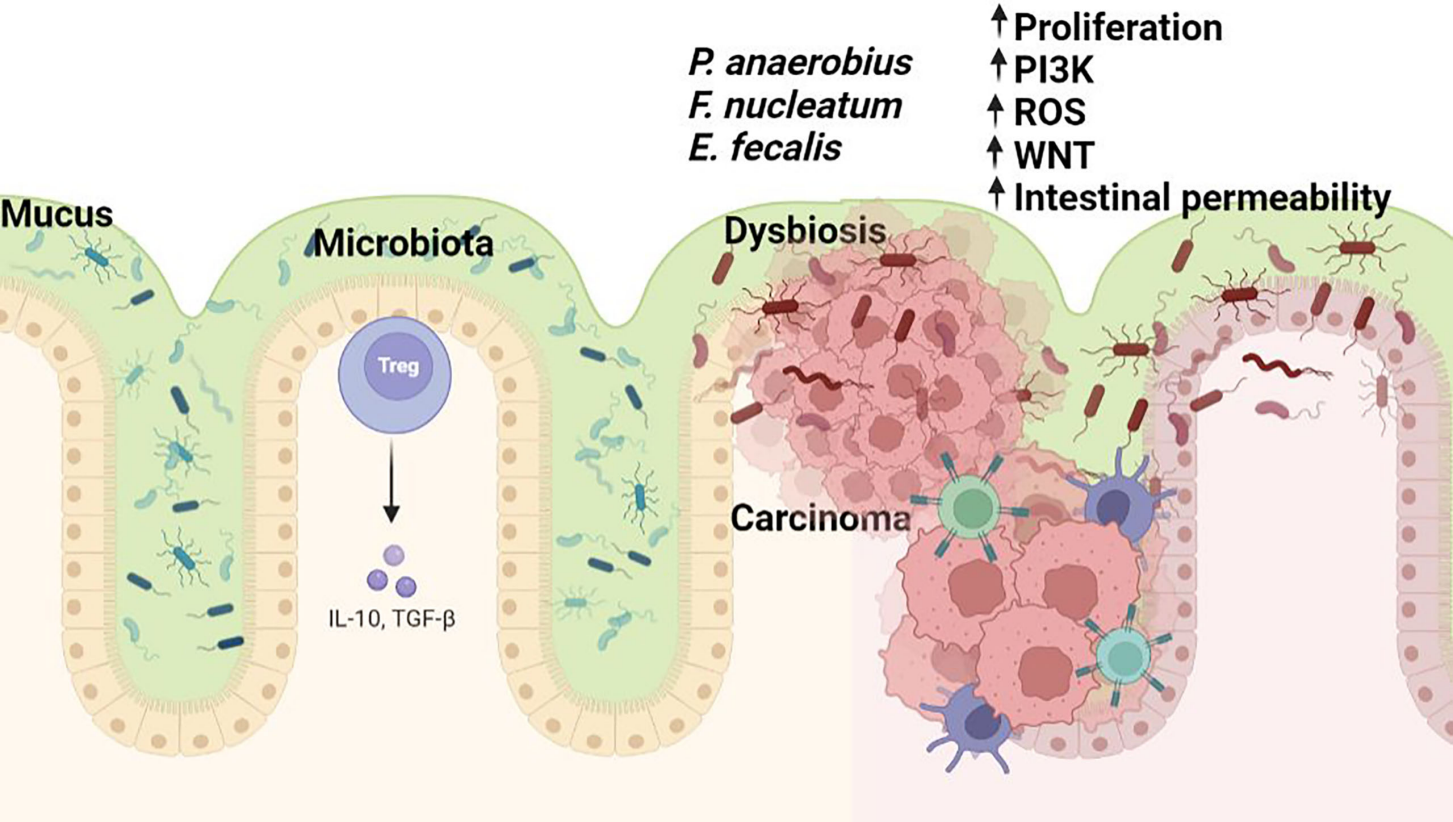
The microbiome in colorectal carcinogenesis. Several bacterial taxa including *Fusobacterium* sp., *Enterococcus* sp., and *P.anaerobius* are commonly associated with colorectal cancer. Dysbiosis hinders the gut barrier function of epithelial tight junctions and the mucus layer favouring exposure of the intestinal epithelium to bacteria and their metabolites (some of which may harbour carcinogenic potential). Bacterial translocation causes enhanced inflammation associated with the production of toxic chemicals or procarcinogen molecules such as reactive oxygen species (ROS) by inflammatory cells (i.e., macrophages). All these changes lead to oxidative and subsequent DNA damage. Figure created in Biorender.com.

Recent mounting evidence suggests that gut microbes can sense certain features of cancer cells which they use for their own advantage. *Fusobacterium nucleatum* interacts with tumor cells *via* its adhesion molecule FadA which binds eukaryotic Annexin A1 in a process mediated by E-cadherin, forming a complex with β-catenin, a central effector of the Wnt pathway (**Figure 1**) (71). *Peptostreptococcus anaerobius*, a microbe enriched in colon tumors can selectively adhere to the CRC mucosa *in vivo*. Due to its surface protein PCWBR2 (putative cell wall binding repeat 2), *P. anaerobius* promotes tumor growth due to activation of the PI3K–AKT pathway and NF-κB-driven inflammation (72).

Importantly, the general conclusion of various studies is that there is not a unique specific microbe that is responsible for CRC, but a group of bacteria whose detrimental actions surpass those of the beneficial microorganisms.

Therefore, to restore the gut eubiosis and achieve a beneficial modulation of the gut microbiome composition and metabolic activities, probiotics might be used to reduce the risk of CRC development. However, although probiotics and prebiotics have shown success in attenuating CRC and its concomitant effects (73), the intimate mechanisms of action in this type of interventional treatments are unknown (74).

## 5 Inflammation as an Earlier Driver of Colorectal Tumorigenesis

Chronic inflammation is one of the hallmarks of CRC and was found to be present from the earliest stages of tumor onset.

### 5.1 Protection Mechanisms Against Gut Inflammation—The Role of Molecular Signaling Crosstalk

Generally, the GI tract has a very good signaling pathway for the rapid activation of anti-inflammatory mechanisms, and the intestinal epithelium is dynamically renewed within a week. This is due to the crypt-progenitor stem cells which can proliferate, differentiate, and are shed at the luminal surface. The intestinal epithelial stem cells generate multiple cell lineages, namely, absorptive enterocytes (80% of the cells), mucus-producing goblet cells (single-cell glands that produce and secrete mucin), enteroendocrine cells, and antimicrobial peptide-producing Paneth cells (75).

The intestinal epithelium provides a dynamic physical barrier that separates the mucosal tissue from pathogens, dietary antigens, and commensal bacteria, and facilitates selective absorption of nutrients, water, and electrolytes. The intestinal epithelial cells (IECs) have specific properties that allow them to manage the complex interaction between the host and the microenvironment, and to maintain the tissue homeostasis. Beside IECs, other types of cells, including immune cells, and the overlaying mucus layer contribute to the protection of the intestinal layer. Damage to the GI barrier leads to homeostasis disruptions, pathological inflammatory responses, and tumorigenesis.

The organization of the mucus layer varies along the length of the colon. Multiple studies examining mucus properties carried out in both mice and humans describe two mucus layers in the colon that include a firm mucus layer adjacent to the epithelium that is devoid of bacteria, which serves as the primary innate defense barrier, with some bacteria colonizing the thin outer layer, and an inner layer which is largely sterile (76, 77). Membrane mucins (hydrophilic branched glycoproteins) provide a safe epithelial cell barrier while playing an important role in signal transduction (78). The distribution of types of mucin differs from one part of gastrointestinal tract to another, and even if MUC5AC is a component of stomach mucus layer (79), it was observed in the distal colon along with MUC2, the major intestinal mucus layer component, during inflammation associated with ulcerative colitis and adenocarcinomas (80, 81).

The mucous layer serves as the outer-most colonic barrier exposed to pathogens and contains mainly mucin2 (MUC2) secreted largely by the goblet cells. Recent research has shown that in some goblet cells (localized at the colonic crypt entrance) there is an endocytosis of LPS and P3CSK4 TRL-ligands that target TRL-MyD88 signaling, which induce ROS, causing the activation of caspase 1 and 11. Subsequently, this process leads to the Ca^2+^-dependent exocytosis of MUC2 and intercellular signaling connections, prompting the secretion of MUC2 by the adjacent responsive goblet cells (82). It has been shown that MUC2 imprint anti-inflammatory gene markers required for oral tolerance on antigen-presenting cells (APCs). Interfering with the APC and epithelial cell interaction reduces the transfer of goblet cell products to APCs, reducing the induction of mucosal reactions. The terminal differentiation of goblet cells is directly controlled by the transcription factor SAM pointed domain-containing ETS transcription factor (Spdef) (83) and also *via* a network of transcriptional factors regulated by the Notch, Wnt/β-catenin, PI3-kinase/Akt and bone morphogenetic protein (BMP) signaling pathways known to influence developmental and inflammation pathways (84). Studies have demonstrated that the IL-6 mediated Jak/STAT3 pathway may drive goblet cells differentiation *via* its downstream PI3-kinase/Akt signal peptide corroborating the previous finding of visible damage of mucosa in IL-6^−/−^ mice (85, 86).

According to Miller, consensus molecular subtype 3 (CMS3) CRC tumors and cell lines are enriched for the expression of goblet cell marker genes. Furthermore, these CMS subtype tumors are mutually exclusive from mucinous adenocarcinoma pathologies (87).

Paneth cells are fully differentiated cells that maintain asepsis of intestinal crypts (88) by secreting the several anti-microbial molecules like defensin-5 (DEFA5) and defensin-6 (DEFA6) and different enzymes, namely, lysozymes and phospholipase A2. Paneth cells are present in chronic non-neoplastic conditions such as inflammatory bowel diseases, and also in neoplastic conditions such as adenoma or carcinoma. The prevalence of Paneth cell differentiation in adenomas varies from 0.2 to 70% (89). In the case of an inflammatory process, Paneth cells occur and tend to linger on after the inflammation was resolved and the crypt structure improved. In the GT, expression of sPLA2-IIA has been localized in Paneth cells of the small intestine, metaplastic Paneth cells of gastric and colonic mucosa, and also columnar epithelial cells of inflammated colonic mucosa (90).

**Figure 2:**
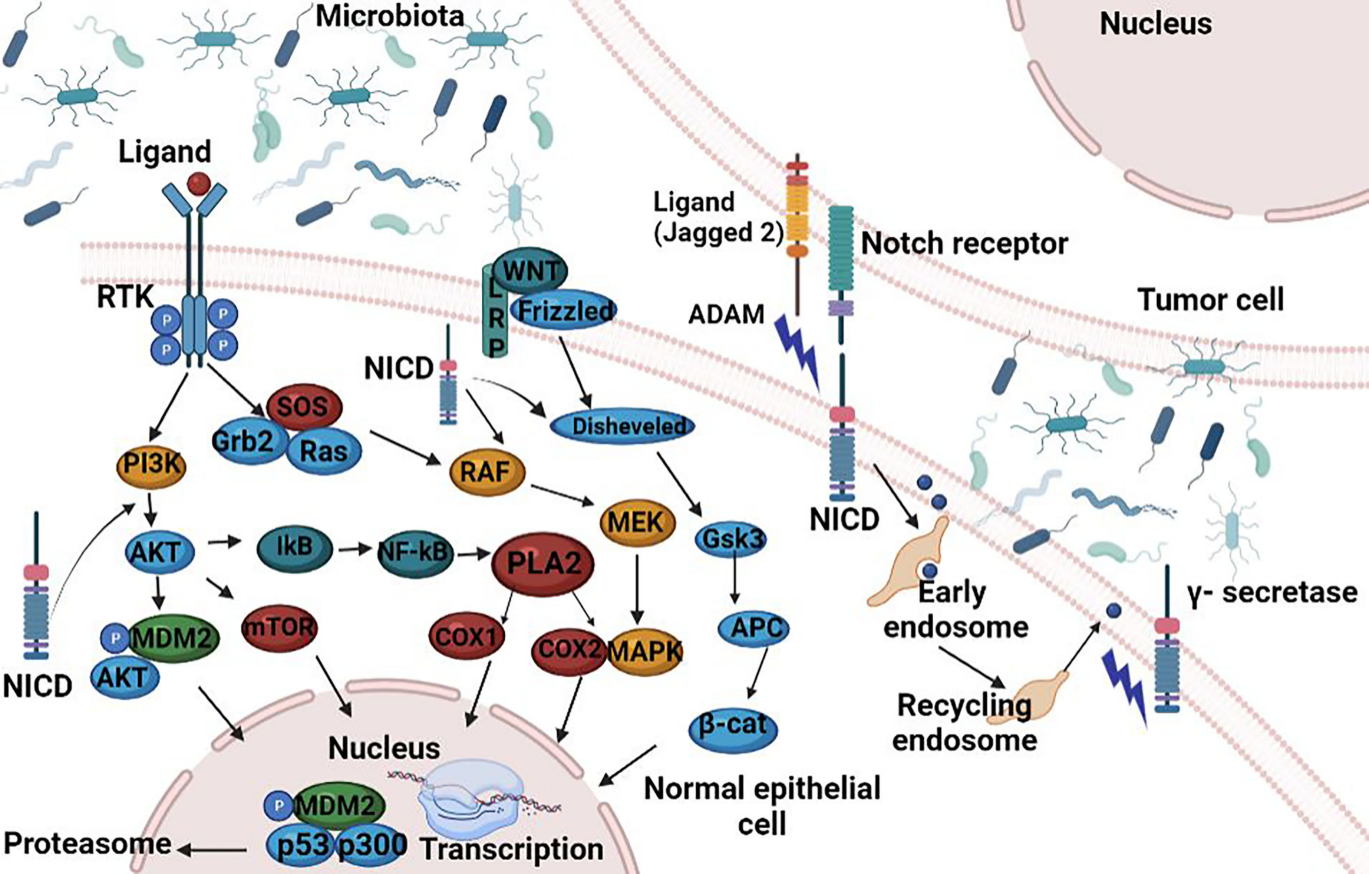
Functional activities of β-catenin. (1) activation of the APC; (2) involvement of the APC in the E-catenin unit (ECCU); (3) activation of the β-catenin degradation site at the C-terminal end of the APC; (4) binding of the APC to the filaments of tubulin; (5) binding of APC to DLG/EB1 proteins. When β-catenin accumulates in the cytosol, the Wnt signalling pathway is inhibited and GSK3β signalling pathway is activated. Active GSK3β simultaneously phosphorylates β-catenin and APC, which it also activates. The APC phosphorylation is possible only if Axin protein is included in the β-catenin-GSK3β-APC assembly also. Once activated, the APC protein is capable to bind free cytoplasmatic β-catenin. APC, “*adenomatous polyposis coli*”; GSH3β, glycogen synthase kinase 3β; DLG and EB1, tumor suppressor proteins; CRD, cysteine-rich domain; Frzb, Frizzby protein; Frizzled, Wnt protein receptor; Dsh, Dishevelled protein; DLG, protein “*human large disc*”; EB1, protein EB1. Figure created in Biorender.com.

Another defensive barrier is represented by the junctions between epithelial cells, namely, tight junction (TJ), adhesion junction (AJ), desmosomes connection, and gap junction from top to basement, (86, 91–93). The TJ present in the cardinal position is composed of occludin, claudins, junctional adhesion molecule (JAM), and zonula occludens (ZO) and limits the passage of macromolecules and microorganisms (91). Early mutagenic events, including loss of adenomatous polyposis coli (APC) and/or activation of β-catenin signaling, may also alter MUC2 and tight junctional proteins and allow for infiltration of pro-tumorigenic microbial products (76). The AJ is an ancient junctional complex that initiates and maintains epithelial cell–cell contacts and has E-cadherin (CDH1) as key transmembrane protein, which mediates calcium-dependent homotypic intercellular adhesions. On the cytoplasmic face of the AJ, E-cadherin associates with p120 (CTNND1), β-catenin (CTNNB1), and α-catenin (CTNNA1), forming a complex that is anchored to cortical actin filaments (75, 94) (**Figure 2**). The assembly and stability of TJ and AJ are maintained by a common regulatory mechanism that involves interactions with the cortical actin cytoskeleton. The cytosolic plaque of TJ and AJ contain actin-binding proteins like α-actin, vinculin, ZO family, afadin, and cingulin that anchor the junctional complexes to F-actin bundles. β-Catenin is a member of the cadherin-based cell adhesive complex, which also acts as a transcription factor if the protein is translocated to the nucleus. When it is not bound to E-cadherin and participating in cell-to-cell adhesion, a cytoplasmic degradation complex (consisting of APC, Axin, GSK-3β, and β-catenin) leads to β-catenin phosphorylation and degradation (75). The extracellular region of E-cadherin from the cell surface binds to cadherins present on adjacent cells, whereas its intracellular region contains binding sites which interact with catenin’s and other regulatory proteins. Regulation of the cytoplasmic level of β-catenin is achieved by the opposite action of Wnt and adenomatous polyposis coli (APC)/E-cadherin (**Figure 2**). The APC protein binds β-catenin to the “Arm” repetitive sequences serving as a negative regulator, while Wnt is a positive regulator of the cytoplasmic level of β-catenin (94, 95).

**Figure 3:**
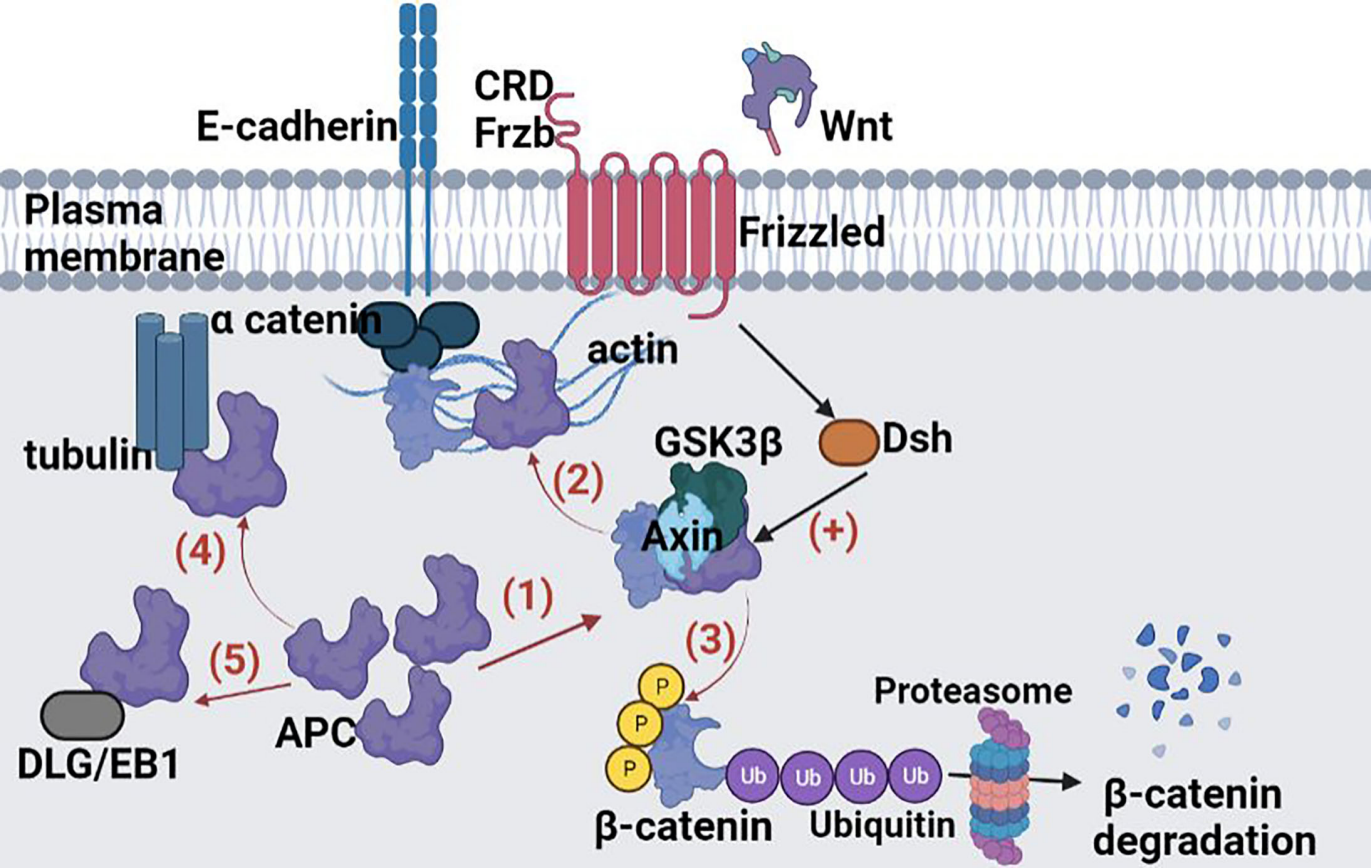
Crosstalk between Notch, Wnt, MAPK and AA signalling pathways. A small increase in cytoplasmic β-catenin levels leads to activation of Wnt gene transcription. The Wnt glycoprotein is synthesized and will be activated by binding to one of the members of the Frizzled family (Frzb)–the Wnt receptor. From the receptor, the signal is transmitted inside the cell to casein kinase II (CKII); it activates and phosphorylates the Disheveled protein (Dsh). The active Disheveled protein is translocated from the cytosol to the cell membrane where, through a signalling process, protein kinase C is phosphorylated, and in turn phosphorylate GSK3β to a N-terminal Ser residue. The effect is the inhibition of GSK3β and finally the β-catenin accumulation in the cytosol. From the cytosol β-catenin is translocated into the nucleus, independent from Lef/Tcf transcription factors translocation. At this level the protein binds Lef/Tcf architectural transcription factors and Wnt gene transcription is induced. Following the interaction with β-catenin, the transcription factors Lef and Tcf can no longer bind to the corresponding sites in the promoter region of some genes and thus the transcriptional process of those genes can take place. In this way Wnt promotes post-transcriptional stabilization and accumulation of β-catenin in the cytosol. Figure created in Biorender.com.

In target cells, the Wnt cellular signaling pathway has the effect of increasing the cytoplasmic level of β-catenin. The Wnt signaling pathway, known as canonical, is one of the major pathways involved in embryogenesis and homeostasis of colorectal tissue, and therefore in carcinogenesis in cases of abnormal activation (96). The role of canonical Wnt signaling in CRC development has been well documented and is presented in **Figure 3**. In the absence of Wnt signals, a multiprotein destruction complex including Axin and the APC facilitates phosphorylation of serine residues in the N terminus of cytosolic β-catenin, which leads to its ubiquitination and proteosomal destruction (95).

The APC signaling pathway has the opposite effect to the Wnt signaling pathway, leading to decreased cytoplasmic β-catenin levels (**Figure 2**).

The deregulation of Wnt signaling is the most frequent molecular aberration in CRC, with inactivating mutations in the APC gene occurring in ∼75% of all tumors (22). The two important partners of Wnt family involved in colorectal carcinogenesis are Wnt5a and Wnt11. On the canonical pathway, after binding to its receptor, extracellular Ca^2+^ initiates the transcription and translation of the canonical Wnt5a in intestinal epithelial cells (97), which upregulates the expression of tumor suppressor 15-PGDH, and further promotes the differentiation of colorectal cancer cells (98). On the noncanonical pathway, Wnt11 enhances the activation of protein kinase C and Ca^2+^/calmodulin-dependent protein-kinase II (CaMKII), which reduces the E-cadherin-mediated cell–cell interaction contact, and further stimulates proliferation and promotes morphological transformation in the intestinal epithelial cells (99). APC encodes a large scaffolding protein that is part of the AXIN destruction complex, which is necessary for the phosphorylation and degradation of β-catenin (100).

Activating mutations in CTNNB1 (β- catenin), or in other Wnt signaling activators (101) can also hyperactivate Wnt signalling in CRC, as can inactivating mutations in Wnt repressors (102). Alternatively, miRNAs can also modulate Wnt signaling through the repression of pathway components. For example, miRNAs correlate with microsatellite instability (MSI) status (103, 104), tumor location (63, 105), BRAF and KRAS mutation (106), (107) and tumor stage (108).

#### 5.1.1 The Antitumorigenic Activity of Phospholipase A2 Type IIA in CRC

Phospholipase A2 type IIA (sPLA2-IIA) is a 14-kDa enzyme found in several tissues and secretory products and is often referred to as an “inflammatory sPLA”. Its expression is induced by pro-inflammatory cytokines and lipopolysaccharides (LPS), and its activity is associated with inflammation, host defence against bacteria, blood coagulation, and atherosclerosis. Many human cells can secrete PLA2G2A, namely, smooth muscle cells, endothelial cells, macrophages, and Paneth cells. Among others, PLA2G2A is involved in arachidonic acid metabolism, antimicrobial activity, exocytosis of endocrine cells, the release of pro-inflammatory mediators, cell proliferation, and cancer. These functions are mediated by both enzyme catalytic and non-catalytic activities of PLA2-IIA. Functional defects in sPLA2-IIA in tumor cells may interfere with the regulatory mechanisms of tumor growth (94). The plasma concentration of sPLA2-IIA increases dramatically in the plasma of CRC patients’ severe infections and other diseases involving generalized inflammation (109), and approximately 55% of CRC patients exhibit high expression of sPLA2-IIA in their epithelial cytoplasm (110). By our studies we indicate that sPLA2-IIA is over-expressed in Paneth cells in adenomas comparative to malignant colorectal tumours, and its PLA2G2A gene is upregulated in human colon adenomas and is frequently subject of loss-of-heterozygosity (LOH) (111, 112). Recently, our results were confirmed by Schewe et al. (17) who provided a potential mechanism by which sPLA2-IIA, expressed by Paneth cells in the small intestine, supresses colon cancer (113). In their studies, Avoranta et al. observed an overexpression of sPLA2-IIA in most colorectal adenomas, where the expression is disturbed, and sPLA2-IIA protein level is dramatically reduced in malignant colorectal tumours as compared to adenomas. In addition, peritumoral mucosa shows increased expression and content of sPLA2-IIA (114). Elevated expression of sPLA2-IIA inhibited colorectal cancer invasion and metastasis through the Wnt/β-catenin signaling pathway (115) and was found to be associated with improved patient survival (116).

The sPLA2-IIA is quickly released (seconds or minutes) from secretory granules following the stimulation of the cell with Ca^2+^ ions or with stimulating agents such as thrombin or ionophores. In contrast to this rapid stage, sPLA2-IIA can also be released after a long period of time (from a few hours to a few days) following the action of some cytokines. The role of these cytokines is to modulate gene expression for sPLA2-IIA, which is why the secretion is delayed.

**Figure 4:**
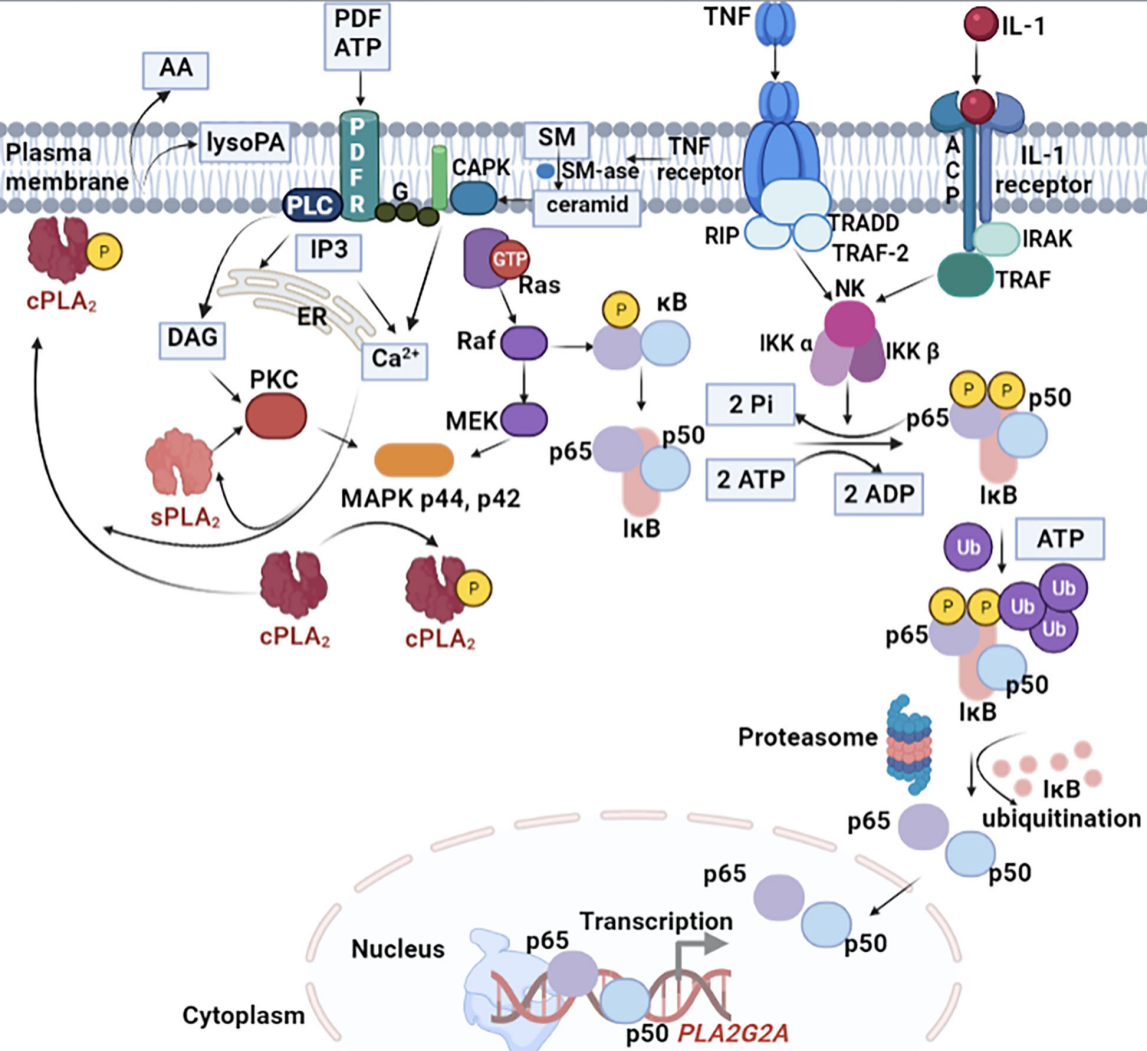
Cellular signalling pathway crosstalk induced by IL-1 for sPLA2-IIA expression. IL-1 induce transcription and secretion of sPLA2-IIA through a mechanism involving the MAPK. A few hours after the enzyme secretion, the signalling pathway is followed by the generation of prostanoids. The induction process is time-dependent and generally occurs after an initial lag period of about 8 h. Both IL-1 and TNF-α stimulate gene transcription and stabilize its RNA. In certain cells, such as for example macrophages, the expression sPLA2-IIA is strongly induced by the two cytokines stimulation under lipopolysaccharides (LPS) action. The MAPK cascade is activated in response to several cytokines, in particular TNF-α and IL-1, and is associated with the activation of nuclear transcriptional factor kB (NF-kB) and several serine/treonin kinases. TNF-α and IL-1 initiates the hydrolysis of sphingomyelin from the plasma membrane. The formed ceramide activates a specific protein kinase (CAPK) on which the MAPK cascade initiation relies on. While the MAPK pathway occurs in the cytosol, the processes that initiate it take place inside the plasma membrane.

The most important cytokines that induce the expression of sPLA2-IIA are IL-1α, IL-1β, and TNF-α. As presented in **Figure 4**, these cytokines induce transcription and secretion of sPLA2-IIA through a mechanism involving the MAP-kinase pathway (MAPK).

Interleukin-6 (IL-6) is a cytokine that recognizes two response elements located in the promoter region of the PLA2G2A gene and induces the expression of the enzyme. The study on hepatomas from the HepG2 cell line revealed that IL-6 induces the expression of both sPLA2-IIA and other acute phase proteins. These observations led the researchers to suggest that sPLA2-IIA is one of the acute phase proteins whose plasma levels increase during the inflammatory response. Il-6 may also induce the expression of sPLA2 type II in human cell lines with invasive gastric cancer.

The most important role of sPLA2-IIA is the release of arahidonic acid (AA) from the structure of membrane phospholipids. The AA concentration inside the cell depends both on the released amount because of the PLA2 action, and on the re-incorporated amount into the structure of the phospholipid following the action of acyltransferase.

#### 5.1.2 The Pro-Inflammatory Activity of Cyclooxygenase-2 in CRC

Cyclooxygenase (COX) enzymes catalyze the conversion of AA, derived from membrane phospholipids by PLA2, into prostanoids which modulate the immune response, blood clotting, and have a role in different pathological conditions, including inflammation.

COX regulates colon carcinoma-induced angiogenesis by two mechanisms: COX-1 regulates angiogenesis in endothelial cells, while COX-2 can modulate production of angiogenic factors by colon cancer cells. While COX-1 is constitutively expressed in many tissues and cell types but in some cases is increased during differentiation, the expression of COX-2 is not usually detectable in normal tissue but is induced by numerous growth factors, hormones, cytokines, and tumor promoters.

COX inhibitors exhibit dramatic antineoplastic activity in several tumor model systems. More than 80% of CRC patients have increased COX-2 levels when compared to adjacent normal tissue. Researchers suggested that COX-2 is expressed in the first steps of colorectal tumorigenesis suggesting an important role of this enzyme in tumor progression. The COX activity is coupled to several terminal synthases that produce five different prostanoids: prostaglandin D2 (PGD2), prostaglandin E2 (PGE2), prostaglandin F2a (PGF2a), prostaglandin I2/prostacyclin (PGI2), and thromboxane A2 (TXA2), some of them also being involved in colon cancer. They have a pro-inflammatory effect, which stimulates cell proliferation, angiogenesis, and resistance to apoptosis. Among the prostanoids, PGE2 has been proposed as the principal prostanoid promoting tumor growth and survival in CRC. PGE2 is present in the healthy colon but its levels are elevated in CRC and correlate with tumour size and progression. More than that, production of PGE2 is induced by COX-2 which in turn increase further expression of COX-2 in colon cancer cells (117, 118). It was observed that COX-2 is stimulated by pro-inflammatory cytokines (TNF, IL1, IL6) produced by the inflammatory cells (119).

#### 5.1.3 NOTCH1 Signaling—A Key in CRC Progression

The Notch signaling pathway significantly regulates the intestinal enterocyte lineage, activating the hairy and enhancer of split 1 (Hes1) transcription factor, and repressing the basic helix-loop-helix (bHLH) transcription factor mouse atonal homolog 1 (Math1), also known as Atonal homologue 1 (Atoh1). Notch signaling pathway activation disrupts the differentiation of secretory cells with the villi coated primarily with absorptive enterocytes associated with Hes1 activation (120). The NOTCH1 cell signaling mechanism is conserved in most multicellular organisms and functions as a receptor for membrane-bound ligands Jagged1, Jagged2, and Delta1 in the regulation of cell-fate determination. According to Schmalhofer et al. (18), the Jagged1-induced Notch1 signalling activation leads to the inhibition of E-cadherin expression, affecting cell–cell adhesion and the simultaneous increase of N-cadherin and vimentin expressions, and nuclear localization of β-catenin (**Figure 2**), which in the end induces an invasive and mesenchymal phenotype (121). Notch signaling has been considered as an oncogene involved in the pathogenesis of CRC (122). In a research published in 2019, Lloyd-Lewis postulates the idea that Notch acts as a biological kapellmeister (orchestra conductor), coordinating spatial cues generated by cell flows during morphogenesis, to dictate cell fate decisions at specific developmental times (123). Earlier studies revealed deregulated Notch signaling in several solid human tumors including CRC. The Notch pathway, is a short-range communication system in which contact between a cell expressing a membrane-bound ligand and a cell expressing a transmembrane receptor initiate a regulatory signal which is sent through a cascade of transcriptional regulatory events that affects the expression of a wide number of genes, resulting in remarkable cell-context dependent pleiotropic effects. Notch proteolysis is required for downstream signaling (124).

Notch also acts as a molecular bridge between stem cells and their non-cellular microenvironment. There is a lot of evidence indicating a bidirectional intercellular communication involving Notch signals between tumor cells and stromal cells in some malignancies (125). Alterations of several signaling pathways, which are correlated with Notch canonical pathway, were described in solid tumors associated with growth activation, resistance to apoptosis, angiogenesis, and invasion/metastatic behavior, but nothing was described related to Notch non-canonical pathway.

In macrophages, the Notch pathway can be activated by Notch ligands that are expressed by macrophages and by Notch ligands expressed on epithelial cells, stromal cells at inflammatory sites or intestinal stem cells. Inflammatory cytokines like TNF and IL1β serve as Notch activators: TNF induces expression of Notch1, Notch4, and Jagged2 and also, the NICD nuclear translocation (126), while IL1β, another important proinflammatory cytokine, induce Notch target genes Hes 1 and Hey1 (127) expression.

At the same time, it was shown that NF-kB signaling interacts with the Notch pathway in many pathologies, including cancer (128). Another group of signaling molecules involved in mediating Notch activation are mitogen-activated protein kinases (MAPKs), a family of serin/threonine protein kinases that are key regulators in inflammation (129).

## 6 Conclusions

An improved approach to cancer biology is expected to explain the reasons for therapeutic failures in the group of patients with early-stage colorectal carcinomas. Colorectal cancer develops because of multiple sequential steps due to the accumulation of genetic alterations including mutations, gene amplification, and epigenetic changes.

To allow survival, evolution has created a robust system of cell-fate regulation that is highly resistant to the loss of one or even a few components. Unfortunately, uncontrolled endogenous and exogenous factors may disturb the regulation system and abnormal cells with abnormal growth mechanisms can develop and modify the normal homeostasis of the body. In CRC specifically, the cellular regulation from the epithelial layer is even more complex due to the presence of microbiota and metabolites resulting from the degradation of nutrients in the daily diet. Therefore, a deeper understanding of the functional roles of the gut microbiome and its interactions with the human host is needed to enable the application of microbiome knowledge to the clinics. As suggested by several authors, imbalances in the normal content of the gut microbiome led to colonization of driver bacteria that induce a chronic inflammation of the colonocytes. This inflammation changes the microenvironment and allows the colonization by passenger bacteria, which may contribute to carcinogenesis process from adenomatosis to tumor formation. So far, there is no universal specific microbiome signature associated with CRC, but the present findings suggest that microbiome could add important information for improving patients’ stratification, providing future leads for preventing CRC tumorigenesis and optimizing CRC therapies through personalized treatment decisions. Analyzing how they communicate with the main signalling pathways involved in carcinogenesis and which is the relationship between different subtypes of tumor cells, microbial signatures and hot response is one of the researchers’ goals for stratifying patients according to the specific crosstalk patterns.

Generally, the GI tract has a very good signalling pathway for a rapid activation of anti-inflammatory mechanisms, and the intestinal epithelium is dynamically renewed within a week. In inflammation processes, the tumor stromal-inflammatory interface represents a dynamic space where growth factors, cytokines, chemokines, enzymes, matrix metalloproteinases, ECM proteins, and metabolic intermediates communicate and facilitate the transition of normal epithelial cells to cancer cells. Further, the cell growth is disrupted, the mutation rate increases, more errors are accumulating in the lipid and glycoprotein biosynthesis, while amino acid supplies decrease. The complex crosstalk between different regulatory pathways allows the tumoral cell to avoid the blocked path and to reroute the signalling network. Moreover, the cell-fate control systems are not only interconnected but also highly redundant, such that if a gene or protein is disabled, another can perform a similar function. Therefore, the system can reset itself to a new status and a new population of cancer cells with new adaptative strategies to the variation of TME conditions is developed. The MSI model (CMS1) became a key biomarker for the early diagnosis and treatment CRC patients, unveiling different microsatellite statuses, compositions and distributions of immune cells and cytokines within TMEs. Unfortunately, the TME versatility hijacked by cancer cells turns the search for a specific drug into a difficult task. Moreover, inhibition of one of specific targets leads to different answers, at different levels, and in different regulatory pathways.

The variability in clinical presentation, aggressiveness, and patterns of treatment failure suggest the necessity of establishing distinct phenotypes-genotypes correlations that can help future treatment strategies. To predict response to therapy, the molecular tests which are currently in use include microsatellite instability (MSI) testing to detect inheritable disease, APC, KRAS, BRAF and other mutational analyses (**Table 2**). In this respect, for the prevention and early diagnosis of CRC, among other currently explored CRC biomarkers, diagnosis must also focus on specific inflammatory markers such as sPLA2-IIA, COX-2, PGE2, β-catenin, or NOTCH1, presented in **Table 3**.

**Table 2:** Biochemical markers used for colorectal cancer screening, diagnosis, and assessment of treatment efficacy.

Market	Targets	Samples	References
**Screening markers**				
gFOBT (Guaiac fecal occult blood test)	Hemoglobin	Stool	(130, 131)
FIT (fecal immunochemical testing)	Hemoglobin	Stool	(132)
MT-sDNA	NDRG4 and BMP3 DNA methylation, haemoglobin	Stool	(133)
DNA methylation	SEPT9 DNA methylation	Blood	(134)
	BCAT1/IKZF1	Blood	(135)
	VIM (vimentin)	Stool	(136)
microRNA (miRNA)	mRNA 7-gene panel	Blood	(137)
	miRNA 5-gene panel	Blood	(138)
	lncRNA 1-gene	Blood	(138)
**Diagnostic Procedures**				
Colonoscopy			(139)
CT colonography			(140, 141)
**Tissue biomarker**				
Immunohistochemistry	Cytokeratins (CKs)	Tissue	(142)
	Caudal type homeobox 2 (CDX2)	Tissue	(143)
	Special AT-rich sequence binding protein2 (SATB2)	Tissue	(144)
	Cadherin 17 (CDH17)	Tissue	(145, 146)
	Telomerase	Tissue	(147, 148)
	GPA33 (A33)	Tissue	(149)
**Predictive and prognostic biomarkers**				
BRAF	Mutations	Blood	(150, 151)
KRAS	Mutations	Blood	(150, 152)
APC	Mutations	Blood	(153)
PIK3CA	Mutations	Blood	(151)
TP53	Mutations	Blood	(154)
NDST4	Allelic imbalance	Blood	(155)
IGFR-1R	Super-expression	Blood	(156)
	Microsatellite instability (MSI) high	Blood	22, 157)

**Table 3:** Specific inflammatory markers that can be used in early-stage colorectal cancer diagnosis.

Markers	Function/Role	Detection Method	Samples	References
COX-2 (Cyclooxygenases)	Significantly promote development and progression of colorectal cancer	Western blot/enzyme immune assay	Cells line	(158)
sPLA2-IIA	Enhances proliferation	IHC	Tissue	(109)
sPLA2-III	Production of pro-inflammatory/pro-tumorigenic lysophosholipids	IHC	Tissue	(159)
Mucin 2	Tumor progression and spread.	IHC	Tissue	(160)
PGE2 pathway	Promoting colorectal tumor growth	IHC/western blot	Cells	(161)
TNF	Development and prognosis of CRC	Meta-analysis	Serum	(162)
NFkB signaling	Promoter of inflammation	Meta-analysis	Serum	(163)
HGF (Hepatocyte growth factor)	Progression and metastasis of colorectal cancer (CRC).	Meta-analysis	Serum	(164)
IGF (Insulin-like growth factors)	Development and progression of several cancers	Meta-analysis	Serum	(165)
NGF (Nerve growth factor)	Proliferation, differentiation and migration of tumor cells	Western blot/elisa	Tissue/serum	(166)
EGF (Epidermal growth factor)	Chemoattractants for endothelial cells	Elisa/IHC	Serum/tissue	(167)
FGF2 (Fibroblast growth factor 2)	Mediates tumor growth	IHC	Tissue	(168)
VEGF (Vascular endothelial growth factor)	Progression and metastases of CRC	IHC	Tissue	(169)
PDGF (Platelet derived growth factor)	Tumor growth and spread	Meta-analysis	Serum	(170)
CCL2 chemokine (C-C motif) ligand 2	Recruitment of monocytes and macrophages	IHC, WB	Tissue	(171)
CCL20 Chemokine (C-C motif) ligand 20	Tumor progression	IHC/Elisa	Tissue/Serum	(172)
CXCL1-CXCL12	Neutrophil’s recruitment	IHC	tissue	(173)
IL-1 (Interleukin-1)	Proinflammatory cytokine	Multiple methods	Serum	(174)
IL-6 Interleukin-6)	Proinflammatory cytokine	Multiple methods	Serum	(175)
IL-8 (Interleukin-8)	Proinflammatory cytokine	Multiple methods	Serum	(176)
MMP-1, (Collagenase-*1)*	Promote the proliferation, migration and invasion of cancer; ECM degradation	Western blot/IHC	Tissue	(177)
MMP-2 (Gelatinase A)	Epithelial-mesenchymal transformation ECM degradation	Western blot/IHC	Tissue	(178)
MMP-7 (Matrilysin)	Cancer progression; Epithelial-mesenchymal transformation	Western blot/IHC	Tissue	(179)
MMP-9 (Gelatinase B)	Degradation of extracellular matrix and regulation of neutrophil action	Western blot/IHC	Tissue	(180)
MMP-13 (Collagenase 3)	Degradation of the extracellular matrix and basement membranes,	Western blot/IHC	Tissue	(181)
MMP-14	CRC progression and prognosis	Western blot/IHC	Tissue	(182)

In the future, the complex network signaling pathway crosstalk between colonocytes, microbiome, and tumor cells at the earlier-stage sporadic colorectal cancer may be of interest for pharmaceutical and bio-pharmaceutical companies in developing target plasma biomarkers, that can be used in screening programs and for establishing phenotype/genotype signatures. Further on, these could help define combinatory strategies to enhance and improve therapies.

## Author Contributions

EI designed the paper structure, performed the literature search, wrote the first draft of the manuscript, and prepared one figure. EI, GG-P, MT, and C-MC wrote sections of the manuscripts. GG analyzed the literature data and prepare the tables. C-MC and GG-P contributed to the design of the manuscript structure. GG-P prepared three pictures. All authors contributed to the article and approved the submitted version.

## Conflict of Interest

The authors declare that the research was conducted in the absence of any commercial or financial relationships that could be construed as a potential conflict of interest.

## Publisher’s Note

All claims expressed in this article are solely those of the authors and do not necessarily represent those of their affiliated organizations, or those of the publisher, the editors and the reviewers. Any product that may be evaluated in this article, or claim that may be made by its manufacturer, is not guaranteed or endorsed by the publisher.
